# Complete Genome Sequence of Classical Swine Fever Virus Genotype 2.2 Strain Bergen

**DOI:** 10.1128/genomeA.00483-14

**Published:** 2014-05-29

**Authors:** Ulrik Fahnøe, Louise Lohse, Paul Becher, Thomas Bruun Rasmussen

**Affiliations:** a DTU National Veterinary Institute, Technical University of Denmark, Lindholm, Kalvehave, Denmark; b EU and OIE Reference Laboratory for Classical Swine Fever (EURL), Institute of Virology, Department of Infectious Diseases, University of Veterinary Medicine, Hannover, Germany

## Abstract

The complete genome sequence of the genotype 2.2 classical swine fever virus strain Bergen has been determined; this strain was originally isolated from persistently infected domestic pigs in the Netherlands and is characterized to be of low virulence.

## GENOME ANNOUNCEMENT

Classical swine fever virus (CSFV) belongs to the genus *Pestivirus* within the family *Flaviviridae*. CSFV is an important animal pathogen that causes disease in pig species and has low-, moderate-, and high-virulence characteristics (1). The CSFV genome consists of a positive-sense RNA, approximately 12.3 kb in length, which encodes a single polyprotein that is co- and posttranslationally cleaved to form the mature structural and nonstructural proteins. The CSFV strains can be divided into genotypes 1, 2, and 3, each comprising three to four subgenotypes (2, 3). CSFV strain Bergen represents a low-virulence strain that originally was isolated from persistently infected pigs in the Netherlands (4). The low-virulence phenotype of the Bergen strain was confirmed in a recent pathogenicity study (5). The Bergen strain has been grouped together with genotype 2.2 strains based on partial 5′-untranslated region (UTR) and E2 sequences (2, 3). Complete genomic sequences have been described for most CSFV genotypes. However, complete genome sequences from genotype 2.2 are lacking in the public sequence databases.

Here, we describe the complete genome sequence of the CSFV genotype 2.2 strain Bergen (isolate CSF0906) obtained from the CSFV collection at the EU Reference Laboratory (EURL). Viral RNA was extracted from infected PK15 cells, and full-length viral cDNAs were amplified by long reverse transcription-PCR (RT-PCR), as previously described (6), using cDNA primer 5′-GGGCCGTTAGGAAATTACCTTAGT- 3′ and PCR primers 5′-TCTATATGCGGCCGCTAATACGACTCACTATAGTATACGAGGTTAGTTCATTCTCGTGTACAATATTGGACAAACTAAATTCAGATTTGG-3′ and 5′-ATATGCGGCCGCGGGCCGTTAGGAAATTACCTTAGTCCAACTAT-3′. The sequencing library was generated from the RT-PCR product using the Ion Plus fragment library kit and sequenced using an Ion Torrent PGM (Life Technologies). Newbler (Roche) was used for *de novo* assembly and the Burrows-Wheeler Aligner (BWA) (7) for mapping of the reads using the *de novo* assembly as the reference sequence. Finally, consensus sequences were aligned using MAFFT in the Geneious software platform (Biomatters).

The final 12,295-nucleotide (nt)-long consensus sequence for the CSFV strain Bergen genome was obtained from a *de novo* assembly consisting of 16,318 sequence reads, with an average sequence depth of 268 reads per nt. The polyprotein-coding sequence is 11,697 nt long and contains 3,899 codons. The 5′ and 3′ UTRs are 372 and 226 nt long, respectively. A comparison with the previously published partial sequence (accession no. JQ411587, 3,508 nt) (3) comprising a part of the 5′ UTR and the coding sequence for the N-terminal autoprotease Npro, capsid protein C, envelope glycoproteins Erns, E1, E2, and the N-terminal part of p7 revealed 7 nt differences. These differences were all mapped to quasispecies populations in the deep sequencing data (data not shown). A comparison with a partial NS5B coding sequence (accession no. AF182909, 409 nt) revealed 100% identity. The complete genome sequence of CSFV strain Bergen should allow for further studies on the genetic diversity and the relationship between the CSFV genotype 2.1 and 2.2 strains.

### Nucleotide sequence accession number. 

The genomic sequence of CSFV strain Bergen has been deposited in GenBank under the accession no. KJ619377.

## References

[1] Floegel-Niesmann G Blome S Gerss-Dülmer H Bunzenthal C Moennig V 2009 Virulence of classical swine fever virus isolates from Europe and other areas during 1996 until 2007. Vet. Microbiol. 139:165–169. 10.1016/j.vetmic.2009.05.008 19576704

[2] Paton DJ McGoldrick A Greiser-Wilke I Parchariyanon S Song JY Liou PP Stadejek T Lowings JP Björklund H Belák S 2000 Genetic typing of classical swine fever virus. Vet. Microbiol. 73:137–157. 10.1016/S0378-1135(00)00141-3 10785324

[3] Postel A Schmeiser S Bernau J Meindl-Boehmer A Pridotkas G Dirbakova Z Mojzis M Becher P 2012 Improved strategy for phylogenetic analysis of classical swine fever virus based on full-length E2 encoding sequences. Vet. Res. 43:50. 10.1186/1297-9716-43-50 22676246 PMC3403906

[4] Van Oirschot JT Terpstra C 1977 A congenital persistent swine fever infection. I. Clinical and virological observations. Vet. Microbiol. 2:121–132. 10.1016/0378-1135(77)90003-7

[5] Lohse L Nielsen J Uttenthal A 2012 Early pathogenesis of classical swine fever virus (CSFV) strains in Danish pigs. Vet. Microbiol. 159:327–336. 10.1016/j.vetmic.2012.04.026 22608103

[6] Rasmussen TB Reimann I Uttenthal A Leifer I Depner K Schirrmeier H Beer M 2010 Generation of recombinant pestiviruses using a full-genome amplification strategy. Vet. Microbiol. 142:13–17. 10.1016/j.vetmic.2009.09.037 19836906

[7] Li H 2013 Aligning sequence reads, clone sequences and assembly contigs with BWA-MEM. arXiv:1303.3997v2. http://arxiv.org/abs/1303.3997v2

